# *Helicobacter pylori* in Vegetables and Salads: Genotyping and Antimicrobial Resistance Properties

**DOI:** 10.1155/2014/757941

**Published:** 2014-08-12

**Authors:** Emad Yahaghi, Faham Khamesipour, Fatemeh Mashayekhi, Farhad Safarpoor Dehkordi, Mohammad Hossein Sakhaei, Mojtaba Masoudimanesh, Maryam Khayyat Khameneie

**Affiliations:** ^1^Baqiyatallah University of Medical Sciences, Tehran, Iran; ^2^Young Researchers and Elites Club, Islamic Azad University, Shahrekord Branch, P.O. Box 166, Shahrekord, Iran; ^3^Intensive and Critical Care Nursing, Jiroft University of Medical Sciences, Jiroft, Iran; ^4^Scientific Association of Veterinary Office, College of Veterinary Medicine, Islamic Azad University, Shahrekord Branch, P.O. Box 166, Shahrekord, Iran; ^5^Department of Gynecology, Imam Reza Hospital, AJA University of Medical Sciences, Tehran, Iran

## Abstract

From a clinical and epidemiological perspective, it is important to know which genotypes and antibiotic resistance patterns are present in* H. pylori* strains isolated from salads and vegetables. Therefore, the present investigation was carried out to find this purpose. Three hundred eighty washed and unwashed vegetable samples and fifty commercial and traditional salad samples were collected from Isfahan, Iran. Samples were cultured and those found positive for *H. pylori* were analyzed using PCR. Antimicrobial susceptibility testing was performed using disk diffusion method. Seven out of 50 (14%) salad and 52 out of 380 (13.68%) vegetable samples harbored* H. pylori*. In addition, leek, lettuce, and cabbage were the most commonly contaminated samples (30%). The most prevalent virulence genes were *oipA* (86.44%) and *cagA* (57.625). *VacA s1a* (37.28%) and *iceA1* (47.45%) were the most prevalent genotypes. Forty different genotypic combinations were recognized. S1a/cagA+/iceA1/oipA+ (33.89%), s1a/cagA+/iceA2/oipA (30.50%), and m1a/cagA+/iceA1/oipA+ (28.81%) were the most prevalent combined genotypes. Bacterial strains had the highest levels of resistance against metronidazole (77.96%), amoxicillin (67.79%), and ampicillin (61.01%). High similarity in the genotyping pattern of* H. pylori* among vegetable and salad samples and human specimens suggests that vegetable and salads may be the sources of the bacteria.

## 1. Introduction

Vegetables are raised as complete foods. Their high values for minerals and vitamins are undeniable and, in a day, millions of people use the vegetables and salads in their main diet. Therefore, hygienic quality of vegetables and salad has a high importance in public health but sometimes it will be changed and several infections and illnesses will occur. Vegetables are in close contact with soil, animal manure, and even human stool. They are usually irrigated with polluted water. Previous studies showed that soil [1], water [2], animal manure [3, 4], and human stool [5, 6] are the main resources for* Helicobacter pylori* (*H. pylori*). Therefore, vegetables can easily be contaminated with* H. pylori*. In addition, their cross-contamination in processing stages is irrefutable.

*H. pylori* is a gram-negative, spiral-shaped bacterium. Its main reservoir is human, particularly the human stomach. It colonizes most of the population, making it one of the most controversial bacteria in the world.* H. pylori* causes peptic ulcer, duodenal ulcer, gastritis, lymphoma, and gastric cancer [7]. According to the reports, the main routes of infection have not been clarified yet [8, 9]. However, it is likely that* H. pylori* infection occurs during childhood or adolescence in both developing and developed countries [8, 9] and its transmission occurs by person to person, either by fecal-oral or oral-oral routes [1]. Nearly 50% of the world population is estimated to be infected with* H. pylori* [10]. The prevalence of this bacterium among Iranian people is 60–90%, indicating that Iran is a high risk region for* H. pylori *infection [11].

Some of the most important virulence factors such as vacuolating cytotoxin A (*vacA*), cytotoxin associated gene (*cag*), induced by contact with the epithelium antigen (*iceA*), outer inflammatory protein (*oipA*), and urease (*ureC*) play a major role in pathogenicity of* H. pylori* infection [12]. These genes are usually induced by adhesion to and invasion of the gastric epithelial cells [13–15]. Genotyping using these well-known virulence marker genes is considered as one of the best approaches for study of correlations between* H*.* pylori *isolates from different samples [16, 17]. The* vacA* gene has a mosaic structure comprising allelic variations in the signal (*s*) and mid region (*m*), each having two different alleles (s1/s2, m1/m2) with different biological activities. Several subregions including s1a, s1b, and s1c and m1a and m1b have been identified in s1 and m1 regions, respectively [18]. Strains carrying the s1m1 mosaic combination of the gene* vacA* exhibit higher levels of cytotoxic activity than s1m2 strains, while s2m2 strains do not secrete the vacuolating cytotoxin [18]. The* iceA* gene has two main allelic variants,* iceA1* and* iceA2,* but their functions are not yet clear. The* cag* pathogenicity island (PAI) has been shown to be involved in inducing ulceration, inflammation, and carcinogenesis [19]. The* cagA* was one of the most common genes in severe cases of peptic ulcer [20]. The* oipA* gene of the* H. pylori* plays an important role in successful colonization of mucosa [21, 22]. The* oipA *gene has the ability to induce interleukin (IL-8) from gastric epithelial cells, as* cagA *and its status have been linked to the discrimination of duodenal ulcer and gastritis [21, 22]. Bacterial urease neutralizes the gastric pH, enabling the colonization of gastric epithelial cells by the bacteria and their motility in the mucus layer [21, 22].

Treatment of diseases caused by* H. pylori* often requires antimicrobial therapy; however, antibiotic-resistant strains of bacteria cause more severe diseases for longer periods of time than their antibiotic-susceptible counterparts. Several studies have shown that antibiotic resistance in* H. pylori* has increased over time [23, 24].

Data on the distribution of genotypes and antibiotic resistance pattern of* H. pylori* strains isolated from vegetable and salad samples are scarce. Therefore, the aim of the present study was genotyping of* H. pylori* strains isolated from vegetable and salad samples and investigating their susceptibility to 13 commonly used antibiotics, as well as investigating seasonal variation in the prevalence of* H. pylori*.

## 2. Materials and Methods

### 2.1. Sample Collection and* H. pylori* Identification

A total of 380 washed and unwashed vegetable samples including leek (*n* = 20), radish (*n* = 20), basil (*n* = 20), parsley (*n* = 20), spinach (*n* = 20), lettuce (*n* = 20), cabbage (*n* = 20), carrot (*n* = 20), scallion (*n* = 20), chive (*n* = 20), fenugreek (*n* = 20), coriander (*n* = 20), pepper (*n* = 20), turnip (*n* = 20), beet (*n* = 20), garlic (*n* = 20), maize (*n* = 20), broccoli (*n* = 20), and cucumber (*n* = 20) and 50 commercial and traditional salad samples were collected from supermarkets and groceries of various parts of Isfahan Province, Iran (Table 2). Samples were collected over a year. Washed vegetables were processed using the high pressure water. All samples were immediately transferred to the Microbiology and Infectious Diseases Research Center of the Islamic Azad University, Shahrekord Branch, at 4°C. Twenty-five milliliters of each homogenized sample was added to 225 mL of Wilkins-Chalgren anaerobe broth (Oxoid, UK) supplemented with 5% of horse serum and colistin methanesulfonate (30 mg/L), cycloheximide (100 mg/L), nalidixic acid (30 mg/L), trimethoprim (30 mg/L), vancomycin (10 mg/L) and colistin methanesulfonate (30 mg/L), cycloheximide (100 mg/L), nalidixic acid (30 mg/L), trimethoprim (30 mg/L), and vancomycin (10 mg/L) and incubated for 7 days at 37°C with shaking under microaerophilic conditions. Then, 0.1 mL of the enrichment selective broth was plated onto Wilkins-Chalgren anaerobe agar supplemented with 5% of defibrinated horse blood and colistin methanesulfonate (30 mg/L), cycloheximide (100 mg/L), nalidixic acid (30 mg/L), trimethoprim (30 mg/L), and vancomycin (10 mg/L) and incubated for 7 days at 37°C under microaerophilic conditions. For comparison, a reference strain of* H. pylori* (ATCC 43504) was employed.

### 2.2. Antimicrobial Susceptibility Testing

Pure cultures of* H. pylori* isolates were used for antibiotic susceptibility test. One strain from each* H. pylori*-positive sample was selected for susceptibility tests. Antimicrobial susceptibility testing was performed by the Kirby-Bauer disc diffusion method using Mueller-Hinton agar (HiMedia Laboratories, Mumbai, India) supplemented with 5% defibrinated sheep blood and 7% fetal calf serum, according to the Clinical Laboratory Standards Institute [25]. The antimicrobial resistance of* H. pylori* was measured against the widely used antibiotics in cases of* H. pylori* gastric ulcer. The following antimicrobial impregnated disks (HiMedia Laboratories, Mumbai, India) were used: metronidazole (5 *μ*g), ampicillin (10 u/), clarithromycin (2 *μ*g), erythromycin (5 *μ*g), tetracycline (30 *μ*g), amoxicillin (10 *μ*g), streptomycin (10 *μ*g), levofloxacin (5 *μ*g), rifampin (30 *μ*g), trimethoprim (25 *μ*g), cefsulodin (30 *μ*g), spiramycin (100 *μ*g), and furazolidone (1 *μ*g). After incubation at 37°C for 48 h in a microaerophilic atmosphere, the susceptibility of the* H. pylori* to each antimicrobial agent was measured and the results were interpreted in accordance with interpretive criteria provided by CLSI (2012) [26]. The* H. pylori* ATCC 43504 was used as control organisms in antimicrobial susceptibility determination.

### 2.3. Detection of* Helicobacter pylori UreB* Gene Using Polymerase Chain Reaction (PCR)

Suspected colonies were identified as* H. pylori* based on the PCR technique. Genomic DNA was extracted from the colonies with typical characters of* H. pylori* using a DNA isolation kit for cells and tissues (Roche Applied Science, Germany, 11814770001) according to the manufacturer's instructions. Set of novel primers for* ureB* gene of the* H. pylori* was designed by the authors. Recorded sequences of the* ureB* gene of the* H. pylori* have been gotten from the GenBank Database of the National Center for Biotechnology Information (NCBI) (GenBank: AY714224.1). The CLS sequence viewer software (Version 6/4) has been used for alignments of the* ureB* gene. Forward and reverse primers have been designed based on the protected area in these sequences. Thermodynamic properties of designed primers were studied using the Gene Runner software (Version 3.05). In order to ensure the specificity of designed primers, the Basic Logical Alignment Search Tool (BLAST) service has been used. The forward primer sequence was* UreB: *5′-CTTAGCGTGGGTCCTGCTAC-3′ and the reverse primer sequence was* UreB: *5′-TGGTGGCACACCATAAGCAT-3′. The gene product was 635 bp. PCR reactions were performed in a final volume of 50 *μ*L containing 5 *μ*L 10 × buffer + MgCl_2_, 2 mM dNTP, 2-unit Taq DNA polymerase, 100 ng genomic DNA as a template, and 25 picomoles of each primer. PCR was performed using a thermal cycler (Eppendorf Co., Germany) under the following conditions: an initial denaturation for 10 minutes at 94°C; 35 cycles for 1 minute at 94°C, 1 minute at 57°C, 1 minute at 72°C, and a final extension at 72°C for 10 minutes. The PCR products were electrophoresed through 1.5% agarose gels (Fermentas, Germany) containing ethidium bromide. A DNA ladder (Fermentas Co., Germany) was used to detect the molecular weight of observed bands under a UV lamp. All tests were performed in triplicate. Samples inoculated with* H. pylori* were used as positive controls.

### 2.4. Genotyping of* Helicobacter pylori*

Presence of the* oipA*,* cagA *and the genotypes of* vacA *(*s1a*,* s1b*,* s1c*,* m1a*,* m1b, *and* m2*) and* iceA* (*iceA1* and* iceA2*) alleles were determined by PCR. The primer sequences are shown in Table 1 [17, 20, 26–30].

The PCR was performed in a total volume of 50 *μ*L containing 1 *μ*M of each primer, 1 *μ*L of genomic DNA (approximately 200 ng), 1 mM of dNTPs mix (Invitrogen), 2 mM of Mgcl_2_, and 0.05 U/*μ*L Taq DNA polymerase (Invitrogen). PCR amplifications were performed in an automated thermal cycler (Biometra Co., Germany). The following cycle conditions were used for PCR amplification: for* vacA*: 32 cycles of 45 s at 95°C, 50 s at 64°C, and 70 s at 72°C; for* cagA*: 1 min at 94°C, 1 min at 56°C, and 1 min at 72°C; for* iceA*: 1 min at 94°C, 1 min at 56°C, and 1 min at 72°C; and, finally, for* oipA*: 1 min at 94°C, 1 min at 56°C, and 1 min at 72°C. All runs included one negative DNA control consisting of PCR grade water and two or more positive controls (26695, J99, SS1, Tx30, 88-23, and 84-183). The amplified products were visualized using ethidium bromide staining after gel electrophoresis of 10 *μ*L of the final reaction mixture in 1.5% agarose.

### 2.5. Statistical Analysis

Data was transferred to Microsoft Excel spreadsheet (Microsoft Corp., Redmond, WA, USA) for analysis. Using SPSS 16.0 statistical software (SPSS Inc., Chicago, IL, USA), Chi-square test and Fisher's exact two-tailed test analysis were performed and differences were considered significant at values of *P* < 0.05. Distribution of genotypes and antimicrobial resistance properties of* H. pylori* isolated from washed and unwashed vegetables and commercial and traditional salads were statistically analyzed.

## 3. Results 

All of the vegetable and salad samples were examined using the culture and PCR techniques. From 380 vegetable and 50 salad samples, 52 (13.68%) and 7 (14%) were positive for* H. pylori*, respectively (Table 2). There were statistically significant differences in the incidence of bacteria in washed and unwashed vegetables and traditional and commercial salad samples (*P* < 0.01). We found that the leek, lettuce, and cabbage samples had the highest incidence of* H. pylori* (Table 2). There were no positive results for pepper, turnip, garlic, chive, and scallion samples. Genotype* oipA* (86.44%) was the most commonly detected genotype in* H. pylori* isolates, followed by* cagA* (57.625) (Table 2). Genotypes* vacA s1a* (37.28%) and* vacA m1a* (30.50%) regions had the highest incidence in* vacA* genotypes, while* vacA s1c* region (10.16%) had the lowest incidence (Table 2). A significant difference was found in the incidence of* oipA* and other genotypes (*P* < 0.05).

Twenty-five and forty-two percent of* H. pylori* strains harbored both m1a and m2, while 22.03% harbored both m1b and m2 (Table 3). Frequency of* cagA*,* oipA,* and both* iceA1* and* iceA2* genotypes was 57.62%, 86.44%, and 40.67%, respectively (Table 3).

Forty different genotypic combinations are shown in Table 4. The most commonly detected combined genotypes were s1a/cagA+/iceA1/oipA+ (33.89%), s1a/cagA+/iceA2/oipA (30.50%), m1a/cagA+/iceA1/oipA+ (28.81%), m1a/cagA+/iceA2/oipA+ (25.42%), and s2/cagA+/iceA1/oipA+ (25.42%).

Descriptions of the seasonal profiles of* H. pylori* isolates are shown in Table 5. Samples which were collected in the spring had the highest incidence (71.18%) of* H. pylori*, while those collected in summer had the lowest incidence (3.38%). There were statistically significant differences (*P* < 0.01) in the incidence of bacteria in spring and other seasons.

Distributions of antimicrobial resistance pattern of* H. pylori* strains are shown in Table 6. The highest levels of antibiotic resistance of the* H. pylori* strains isolated from vegetable and salad samples were found against metronidazole (77.96%), followed by amoxicillin (67.79%) and ampicillin (61.01%). Bacterial strains of our study were susceptible to levofloxacin, rifampin, trimethoprim, cefsulodin, and spiramycin. We found statistically significant differences in the incidence of bacterial antibiotic resistance against metronidazole, streptomycin, furazolidone, and rifampin (*P* < 0.05).

## 4. Discussion

Totally, 13.72% of vegetable and salad samples of our investigation were contaminated with* H. pylori*. High prevalence of* H. pylori* in clinical samples was reported from Scandinavia, Turkey, Japan, Pakistan, South America, and England [31], while low prevalence was reported from Canada [29]. Our work has identified marked seasonality in the incidence of* H. pylori* isolated from vegetable and salad samples.* H. pylori* isolates had the highest incidence in spring season (71.18%). Moshkowitz et al. (1994) [32] reported that the frequency of* H. pylori* infection in dyspeptic patients in Israel is significantly increased in the humid and rainfall months and decreases in the summer, which is similar to our results. Similar seasonal distributions of* H. pylori* were reported previously [33, 34].

Leek, lettuce, and cabbage were the most commonly contaminated samples in our investigation as they are grown in manure rich soil and thus can easily be infected. Differences in amount of activated water (AW), pH, and hygienic conditions during processing of vegetable and salad samples caused high differences in the incidence of* H. pylori* in our study. Also, the role of infected staffs as sources of* H. pylori* infection is so important [11]. The main reason for the high distribution of* H. pylori* in commercial salad samples is the fact that maybe some food safety and quality standards (good agricultural practices (GAPs), good manufacturing practices (GMPs), and the hazard analysis and critical control point (HACCP) system need to be applied and performed in most of the Iranian food units to control growth, proliferation, and survival of bacteria during harvesting, distribution, and storage periods.

High incidence of* H. pylori* in uncooked vegetables that had been irrigated with water contaminated with sewage was reported previously [35, 36]. Frequent consumption of raw vegetables was associated with likelihood of* H. pylori* infection [37]. Also, individuals who consume vegetables are more likely to acquire* H. pylori* [38]. Foods with water activity higher than 0.96 and pH from 4.9 to 9.0 (like vegetables) theoretically provide conditions for the survival of* H. pylori* [39].

The most commonly detected virulence genes in* H. pylori* strains of our study were* oipA* (86.44%),* cagA* (57.62%),* iceA1* (47.45%), and* iceA2* (42.37%). High presence of these genes in clinical samples has been reported previously from Japan [40], Turkey [41], Nigeria [42], and the United States [43]. These virulence genes are responsible for cytotoxin production [44], interleukin-8 (IL-8) construction [45, 46], vacuolization and apoptosis in gastric epithelial cells [13, 14], adhesion to gastric epithelial cells, and inflammatory effects [15, 47].

Alleles* vacA s1a* (37.28%) and* iceA1* (47.45%) were the most commonly detected genotypes in* vacA* and* iceA* positive samples of our study, respectively.* VacA m1a/s1* (27.11%),* vacA m2/s2* (25.42%),* vacA m1a/m2* (25.42%), and* iceA1/iceA2* (40.67%) were the most commonly detected genotypes in our study. There were no previously published data about the genotyping of* H. pylori* in vegetables, salads, and even other types of foods. Various genotypes of* vacA* strains were the most commonly detected genotypes in the studies of Linpisarn et al. (2007) (Thailand) [48], López-Vidal et al. (2008) (Mexico) [49], and Rudi et al. (1998) (Germany) [50]. The high presence of* vacA s1a*/*m2* genotypes has been reported previously from Iran [11] and Germany [50] but far different results have been reported from Thailand [48] and Mexico [49].

Bacterial strains of our study were resistant to the majority of tested antibiotics. We found that bacterial strains exhibited the highest level of resistance to metronidazole (77.96%), amoxicillin (67.79%), ampicillin (61.01%), and tetracycline (59.32%). The high antibiotic resistance to these drugs detected in our study indicates that irregular and unauthorized use of them may have occurred in Iran. Similarly, metronidazole, amoxicillin, ampicillin, and tetracycline resistance profiles have been reported previously [51, 52]. Indian strains of* H. pylori* had the highest antibiotic resistance against metronidazole (77.9%), clarithromycin (44.7%), and amoxicillin (32.8%) [52], which was similar to our results. Bang et al. (2007) [25] found that the* H. pylori* isolates had the high antibiotic resistance to metronidazole (34.7%), clarithromycin (16.7%), and amoxicillin (11.8%). Low antibiotic resistance of* H. pylori* strains against levofloxacin, rifampin, trimethoprim, cefsulodin, and spiramycin may be due to the regular and low prescription of these antibiotics.

*H. pylori* isolates from African countries like Senegal and Nigeria, Asian countries like India, Taiwan, China, Iran, Egypt, Saudi Arabia, and Thailand, and South American countries like Argentina, Brazil, and Colombia had the highest antibiotic resistance to metronidazole, followed by clarithromycin, amoxicillin, quinolones, tetracycline, and furazolidone [30], which was similar to our results.

The above data highlight large differences in the prevalence of* H. pylori* in different studies, as well as differences in virulence genes, genotypes, and antibiotic resistance patterns in the clinical samples. This could be related to differences in the type of sample tested (stool, gastric biopsy, saliva, and food), number of samples, method of sampling, experimental methodology, geographical area, antibiotic prescription preference among clinicians, antibiotic availability, and climate differences in the areas where the samples were collected, which would have differed in each study.

## 5. Conclusions

In conclusion, vegetable and salad samples harbor* H. pylori* similar in genotype of the* vacA*,* cagA*,* oipA,* and* iceA* alleles to isolates recovered from humans. Also, there was a high similarity in the genotyping pattern of* H. pylori* DNA among vegetable and salad samples and human specimens of other investigations suggest that vegetables and salads are the sources of the bacteria and that they entered the human population in a period of time. On the other hand, diversity of* H. pylori *genotypes in vegetable and salad samples with the clinical isolates of other studies suggested that consumption of contaminated vegetables and salads with* H. pylori *strains may be a threat to human health. Our findings should raise awareness about antibiotic resistance in* H. pylori* strains in Iran. Clinicians should exercise caution when prescribing antibiotics, especially during the spring season. Our data showed that conventional ways to wash vegetables cannot reduce their contamination.

## Figures and Tables

**Table 1:** Oligonucleotide primers used for genotyping of *Helicobacter pylori* isolated from vegetables and salads in Iran.

Genes names	Primer sequence (5′-3′)	Size of product (bp)
*ureC *	F∗: GCTTACTTTCTAACACTAACGCGC R∗∗: GGATAAGCTTTTAGGGGTGTTAGGGG	296

*vacA s1a *	F: CTCTCGCTTTAGTAGGAGC R: CTGCTTGAATGCGCCAAAC	213

*vacA s1b *	F: AGCGCCATACCGCAAGAG R: CTGCTTGAATGCGCCAAAC	187

*vacA s1c *	F: CTCTCGCTTTAGTGGGGYT R: CTGCTTGAATGCGCCAAAC	213

*vacA s2 *	F: GCTAACACGCCAAATGATCC R: CTGCTTGAATGCGCCAAAC	199

*vacA m1A *	F: GGTCAAAATGCGGTCATGG R: CCATTGGTACCTGTAGAAAC	290

*vacA m1B *	F: GGCCCCAATGCAGTCATGGA R: GCTGTTAGTGCCTAAAGAAGCAT	291

*vacA m2 *	F: GGAGCCCCAGGAAACATTG R: CATAACTAGCGCCTTGCA	352

*cagA *	F: GATAACAGCCAAGCTTTTGAGG R: CTGCAAAAGATTGTTTGGCAGA	300

*iceA1 *	F: GTGTTTTTAACCAAAGTATC R: CTATAGCCASTYTCTTTGCA	247

*iceA2 *	F: GTTGGGTATATCACAATTTAT R: TTRCCCTATTTTCTAGTAGGT	229/334

*oipA *	F: GTTTTTGATGCATGGGATTT R: GTGCATCTCTTATGGCTTT	401

∗F: forward.

∗∗R: reverse.

**Table 2:** Distribution of *Helicobacter pylori* genotypes isolated from washed and unwashed vegetables and commercial and traditional salads in Iran.

Types and numbers of samples	*Helicobacter pylori * positive (%)	Genotypes (%)										
		*vacA *							*cagA *	*iceA *		*oipA *
		*S1a *	*S1b *	*S1c *	*S2 *	*M1a *	*M1b *	*M2 *		*IceA1 *	*IceA2 *	
Salads												
Traditional (25)	5 (20)	1	1	—	1	2	1	2	4	3	2	5
Commercial (25)	2 (8)	1	—	—	1	1	—	1	2	1	—	2
**Total (50)**	**7 (14)**	**2 (28.57)**	**1 (14.28)**	**—**	**2 (28.57)**	**3 (42.85)**	**1 (14.28)**	**3 (42.85)**	**6 (85.71)**	**4 (57.14)**	**2 (28.57)**	**7 (100)**

Leek												
Washed (10)	1 (10)	1	—	—	—	1	1	—	1	—	1	1
Unwashed (10)	5 (50)	2	1	1	1	1	1	2	3	2	2	4
**Total (20)**	**6 (30)**	**3 (50)**	**1 (16.66)**	**1 (16.66)**	**1 (16.66)**	**2 (33.33)**	**2 (33.33)**	**2 (33.33)**	**4 (66.66)**	**2 (33.33)**	**3 (50)**	**5 (83.33)**

Radish												
Washed (10)	—	—	—	—	—	—	—	—	—	—	—	—
Unwashed (10)	2 (20)	1	—	—	1	1	—	1	1	1	1	1
**Total (20)**	**2 (10)**	**1 (50)**	**—**	**—**	**1 (50)**	**1 (50)**	**—**	**1 (50)**	**1 (50)**	**1 (50)**	**1 (50)**	**1 (50)**

Basil												
Washed (10)	—	—	—	—	—	—	—	—	—	—	—	—
Unwashed (10)	3 (30)	1	1	—	1	1	1	—	2	2	1	3
**Total (20)**	**3 (15)**	**1 (33.33)**	**1 (33.33)**	**—**	**1 (33.33)**	**1 (33.33)**	**1 (33.33)**	**—**	**2 (66.66)**	**2 (66.66)**	**1 (33.33)**	**3 (100)**

Parsley												
Washed (10)	—	—	—	—	—	—	—	—	—	—	—	—
Unwashed (10)	3 (30)	1	1	1	1	1	1	1	2	2	1	2
**Total (20)**	**3 (15)**	**1 (33.33)**	**1 (33.33) **	**1 (33.33)**	**1 (33.33)**	**1 (33.33)**	**1 (33.33)**	**1 (33.33)**	**2 (66.66)**	**2 (66.66)**	**1 (33.33)**	**2 (66.66)**

Spinach												
Washed (10)	1 (10)	1	1	—	—	—	1	—	1	1	1	1
Unwashed (10)	4 (40)	1	1	1	1	1	1	1	2	2	2	3
**Total (20)**	**5 (25)**	**2 (40)**	**2 (40)**	**1 (20)**	**1 (20)**	**1 (20)**	**2 (40)**	**1 (20)**	**3 (60)**	**3 (60)**	**3 (60)**	**4 (80)**

Lettuce												
Washed (10)	2 (20)	1	—	1	1	—	1	—	1	1	1	2
Unwashed (10)	4 (40)	1	1	1	1	1	1	1	2	2	2	4
**Total (20)**	**6 (30)**	**2 (33.33)**	**1 (16.66)**	**2 (33.33)**	**2 (33.33)**	**1 (16.66)**	**2 (33.33)**	**1 (16.66)**	**3 (50)**	**3 (50)**	**3 (50)**	**6 (100)**

Cabbage												
Washed (10)	2 (20)	1	1	—	1	1	1	1	1	1	2	2
Unwashed (10)	4 (40)	2	1	1	1	1	1	2	2	2	2	3
**Total (20)**	**6 (30)**	**3 (50)**	**2 (33.33)**	**1 (16.66)**	**2 (33.33)**	**2 (33.33)**	**2 (33.33)**	**3 (50)**	**3 (50)**	**3 (50)**	**4 (66.66)**	**5 (83.33)**

Carrot												
Washed (10)	—	—	—	—	—	—	—	—	—	—	—	—
Unwashed (10)	1 (10)	—	—	—	—	—	—	—	—	—	—	1
**Total (20)**	**1 (5)**	**—**	**—**	**—**	**—**	**—**	**—**	**—**	**—**	**—**	**—**	**1 (100)**

Fenugreek												
Washed (10)	1 (10)	—	—	—	—	—	—	—	1	—	—	1
Unwashed (10)	3 (30)	1	1	—	—	1	—	—	2	2	1	3
**Total (20)**	**4 (20)**	**1 (25)**	**1 (25)**	**—**	**—**	**1 (25)**	**—**	**—**	**3 (75)**	**2 (50)**	**1 (25)**	**4 (100)**

Coriander												
Washed (10)	1 (10)	—	—	—	—	—	—	—	1	1	—	1
Unwashed (10)	3 (30)	1	1	—	1	1	1	1	1	1	1	2
**Total (20)**	**4 (20)**	**1 (25)**	**1 (25)**	**—**	**1 (25)**	**1 (25)**	**1 (25)**	**1 (25)**	**2 (50)**	**2 (50)**	**1 (25)**	**3 (75)**

Beet												
Washed (10)	1 (10)	—	—	—	—	—	—	—	—	—	1	1
Unwashed (10)	4 (40)	2	1	—	1	1	1	1	2	2	2	3
**Total (20)**	**5 (25)**	**2 (40)**	**1 (20)**	**—**	**1 (20)**	**1 (20)**	**1 (20)**	**1 (20)**	**2 (40)**	**2 (40)**	**3 (60)**	**4 (80)**

Maize												
Washed (10)	—	—	—	—	—	—	—	—	—	—	—	—
Unwashed (10)	1 (10)	—	—	—	—	—	—	—	—	—	—	1
**Total (20)**	**1 (5)**	**—**	**—**	**—**	**—**	**—**	**—**	**—**	**—**	**—**	**—**	**1 (100)**

Broccoli												
Washed (10)	1 (10)	1	—	—	—	1	—	—	1	—	—	1
Unwashed (10)	2 (20)	1	1	—	1	1	—	—	1	1	1	2
**Total (20)**	**3 (15) **	**2 (66.66)**	**1 (33.33)**	**—**	**1 (33.33)**	**2 (66.66)**	**—**	**—**	**2 (66.66)**	**1 (33.33)**	**1 (33.33)**	**3 (100)**

Cucumber												
Washed (10)	—	—	—	—	—	—	—	—	—	—	—	—
Unwashed (10)	3 (30)	1	1	—	1	1	1	1	1	1	1	2
**Total (20)**	**3 (15)**	**1 (33.33)**	**1 (33.33)**	**—**	**1 (33.33)**	**1 (33.33)**	**1 (33.33)**	**1 (33.33)**	**1 (33.33)**	**1 (33.33)**	**1 (33.33)**	**2 (66.66)**

Total (430)	59 (13.72)	22 (37.28)	14 (23.72)	6 (10.16)	15 (25.42)	18 (30.50)	14 (23.72)	15 (25.42)	34 (57.62)	28 (47.45)	25 (42.37)	51 (86.44)

**Table 3:** Distribution of *Helicobacter pylori* genotypes isolated from vegetables and salad samples in Iran.

Genotypes	Prevalence (%)
*vacA *	
M1as1a	16 (27.11∗)
M1as1b	14 (23.72)
M1bs1a	13 (22.03)
M1bs1b	14 (23.72)
M1as1c	4 (6.77)
M1bs1c	2 (3.38)
M2s1a	14 (23.72)
M2s1b	11 (18.64)
M2s1c	3 (5.08)
M2s2	15 (25.42)
M1as2	13 (22.03)
M1bs2	11 (18.64)
M1am2	15 (25.42)
M1bm2	13 (22.03)

*cagA *	
CagA+	34 (57.62)
CagA−	25 (42.37)

*iceA *	
IceA1	28 (47.45)
IceA2	25 (42.37)
IceA1 IceA2	24 (40.67)

*oipA *	
OipA+	51 (86.44)
OipA−	8 (13.55)

∗Percentage of positive genes from total 59 positive samples.

**Table 4:** Combined *vacA*, *cagA*, *iceA,* and *oipA* genotypes of *Helicobacter pylori* isolated from salads and vegetables in Iran.

Combined genotypes	Total (59∗) (%)
s1a/cagA+/iceA1/oipA+	20 (33.89)
s1b/cagA+/iceA1/oipA+	11 (18.64)
s1c/cagA+/iceA1/oipA+	6 (10.16)
s1a/cagA+/iceA2/oipA+	18 (30.50)
s1b/cagA+/iceA2/oipA+	12 (20.33)
s1c/cagA+/iceA2/oipA+	5 (8.47)
s1a/cagA−/iceA1/oipA+	11 (18.64)
s1b/cagA−/iceA1/oipA+	8 (13.55)
s1c/cagA−/iceA1/oipA+	4 (6.77)
s1a/cagA−/iceA2/oipA+	10 (16.94)
s1b/cagA−/iceA2/oipA+	7 (11.86)
s1c/cagA−/iceA2/oipA+	4 (6.77)
s1a/cagA+/iceA1/oipA−	6 (10.16)
s1b/cagA+/iceA1/oipA−	3 (5.08)
s1c/cagA+/iceA1/oipA−	2 (3.38)
s2/cagA+/iceA1/oipA+	15 (25.42)
s2/cagA+/iceA2/oipA+	14 (23.72)
s2/cagA−/iceA1/oipA+	12 (20.33)
s2/cagA−/iceA2/oipA+	10 (16.94)
s2/cagA−/iceA2/oipA−	6 (10.16)
s2/cagA+/iceA2/oipA−	7 (11.86)
s2/cagA+/iceA1/oipA−	8 (13.55)
m1a/cagA+/iceA1/oipA+	17 (28.81)
m1b/cagA+/iceA1/oipA+	13 (22.03)
m1a/cagA+/iceA2/oipA+	15 (25.42)
m1b/cagA+/iceA2/oipA+	12 (20.33)
m1a/cagA−/iceA1/oipA+	14 (23.72)
m1b/cagA−/iceA1/oipA+	11 (18.64)
m1a/cagA−/iceA2/oipA+	12 (20.33)
m1b/cagA−/iceA2/oipA+	10 (16.94)
m1a/cagA+/iceA1/oipA−	5 (8.47)
m1b/cagA+/iceA2/oipA−	3 (5.08)
m2/cagA+/iceA1/oipA+	14 (23.72)
m2/cagA+/iceA2/oipA+	13 (22.03)
m2/cagA+/iceA2/oipA−	5 (8.47)
m2/cagA+/iceA1/oipA−	6 (10.16)
m2/cagA−/iceA1/oipA+	11 (18.64)
m2/cagA−/iceA2/oipA+	10 (16.94)
m2/cagA−/iceA2/oipA−	3 (5.08)
m2/cagA−/iceA1/oipA−	4 (6.77)

∗Total positive samples.

**Table 5:** Seasonal distribution of *Helicobacter pylori* isolated from washed and unwashed vegetables and commercial and traditional salads in Iran.

Types and numbers of positive samples	Seasonal distribution (%)			
	Winter	Summer	Autumn	Spring
Salads				
Traditional (5∗)	1 (20)	—	1 (20)	3 (60)
Commercial (2)	—	—	—	2 (100)
Total (7)	1 (14.28)	—	1 (14.28)	5 (71.42)

Vegetables				
Washed (10)	2 (20)	—	1 (10)	7 (70)
Unwashed (42)	6 (14.28)	2 (4.76)	4 (9.52)	30 (71.42)
Total (52)	8 (15.38)	2 (3.84)	5 (9.61)	37 (71.15)

Total				
Vegetables and salads (59)	9 (15.25)	2 (3.38)	6 (10.16)	42 (71.18)

∗Numbers of positive samples.

**Table 6:** Distribution of antibiotic resistance properties of *Helicobacter pylori* isolated from salads and vegetables in Iran.

Types and numbers of positive samples	Antibiotic resistance properties (%)												
	METR5∗	AM10	CLRT2	ERT5	TE30	AMX10	S10	FZL1	Lev5	Rif30	TRP25	Cef30	Spi100
Salads													
Traditional (5)	4 (80)	2 (40)	1 (20)	1 (20)	3 (60)	3 (60)	1 (20)	1 (20)	1 (20)	—	3 (60)	1 (20)	1 (20)
Commercial (2)	2 (100)	1 (50)	—	—	1 (50)	1 (50)	—	—	—	—	1 (50)	—	—
Total (7)	6 (85.71)	3 (42.85)	1 (14.28)	1 (14.28)	4 (57.14)	4 (57.14)	1 (14.28)	1 (14.28)	1 (14.28)	—	4 (57.14)	1 (14.28)	1 (14.28)

Vegetables													
Washed (10)	7 (70)	5 (50)	2 (20)	2 (20)	5 (50)	6 (60)	1 (10)	1 (10)	1 (10)	—	5 (50)	2 (20)	1 (10)
Unwashed (42)	33 (78.57)	28 (66.66)	10 (23.80)	11 (26.19)	26 (61.9)	30 (71.42)	7 (16.66)	7 (16.66)	3 (7.14)	2 (4.76)	26 (61.9)	5 (11.90)	3 (7.14)
Total (52)	40 (76.92)	33 (63.46)	12 (23.07)	13 (25)	31 (59.61)	36 (69.23)	8 (15.38)	8 (15.38)	4 (7.69)	2 (3.84)	31 (59.61)	7 (13.46)	4 (7.69)

Total													
Vegetables and salads (59)	46 (77.96)	36 (61.01)	13 (22.03)	14 (23.72)	35 (59.32)	40 (67.79)	9 (15.25)	9 (15.25)	5 (8.47)	2 (3.38)	35 (59.32)	8 (13.55)	5 (8.47)

∗In this table, METR5 = metronidazole (5 *µ*g/disk); AM10 = ampicillin (10 *µ*g/disk); CLRT2 = clarithromycin (2 *µ*g/disk); ERT5 = erythromycin (5 *µ*g/disk); TE30 = tetracycline (30 *µ*g/disk); AMX10 = amoxicillin (10 *µ*g/disk); S10 = streptomycin (10 *µ*g/disk); and FZL1 = furazolidone (1 *µ*g/disk); Lev5 = levofloxacin (5 *µ*g/disk); Rif30 = rifampin (30 *µ*g/disk); TRP25 = trimethoprim (25 *µ*g/disk); Cef30 = cefsulodin (30 *µ*g/disk); and Spi100 = spiramycin (100 *µ*g/disk).
